# Editorial: Recent Advances in Promoters for Gas Hydrate Formation

**DOI:** 10.3389/fchem.2021.708269

**Published:** 2021-06-04

**Authors:** Fei Wang, Amadeu K. Sum, Bei Liu

**Affiliations:** ^1^ Qingdao University of Science and Technology, Qingdao, China; ^2^ Colorado School of Mines, Golden, CO, United States; ^3^ China University of Petroleum, Beijing, China

**Keywords:** gas hydrates, promoters, kinetics, thermodynamics, carbon material

Gas hydrates have been endowed with great potential in gas storage & transportation (e.g. methane and hydrogen), gas separation (e.g. CO_2_ capture), desalination, cold and thermal energy storage, etc. Achieving the rapid hydrate formation is critical for utilizing the hydrate technology, which has been being the research focus since the proposition of this technology. During the past 2 decades, using additives to promote hydrate formation has been confirmed as an efficient method to push forward the application of hydrate technology, usually consisting of kinetic promoters (e.g. surfactants and nanofluids) and thermodynamic promoters (e.g. cyclopentane and tetrahydrofuran). However, more work is necessary to finally bring the hydrate technology from lab-scale to industrial application due to the defects of the current promoters, such as, the foam generation caused by surfactants, the poor stability of nanofluids, etc. On this account, the current research topic is focused on gas hydrate promoters, including both the summaries on the current promoters and the up to date development on novel promoters.

This research topic contains four mini review and five original research articles, covering both kinetic and thermodynamic promoters. Carbon-based materials, including activated carbon, graphite, graphene, carbon nanotube, etc., have been successfully adopted as kinetic promoters for gas hydrate formation. In the mini review by Song et al., the promotion efficiency of above-mentioned materials to gas hydrate formation was summarized and evaluated, in which, the carbon-based materials were applied as three types: porous carbon materials as packed beds, carbon-based particles in suspensions, and carbon-based nanoparticles in nanofluids. Based on the evaluation, porous packed bed (e.g. activated carbon) could promote gas hydrate formation by providing a large gas/liquid interface, and particles in suspension (e.g. activated carbon and carbon nanotubes) and nanofluids (e.g. water-soluble oxidized carbon nanotubes) could contribute to heat and mass transfer during hydrate formation. Among the common carbon-based materials, graphene is the one with the most attention in various fields. In the mini review by Sun et al., graphene-based materials adopted as suspension or nanofluids for promoting gas hydrate formation were specially summarized, including graphene, oxidized/sulfonated graphene, surfactant-stabilized graphene, and graphene-carried metal nanoparticles. No matter which form graphene was used, the hydrate nucleation could be promoted by the presence of numerous nucleation sites and the heat transfer during hydrate formation could be enhanced by the high thermal conductivity of graphene. Activated carbon, due to the abundant pore structures, has also been widely used in promoting gas hydrate formation. Zhang et al., chose activated carbon as the research object and summarized the promotion mechanism of activated carbon as porous packed bed to gas hydrate formation, in which, the hydrate nucleation sites provided by the microbulges on activated carbon surface and the two-way convection of water and methane in micropores of activated carbon were considered as the main reasons of the high hydrate nucleation and growth kinetics. Chen et al.used another porous material, ZIF-8 (2-methylimidazole zinc salt), as both slurry and packed bed for methane hydrate formation and proposed the adsorption-hydration sequence method for high-efficiency methane storage with ZIF-8 slurry as the medium. Moreover, with the increasing severity of environmental problems, environment-friendly materials for gas hydrate formation have attracted more and more attention. Zhang et al. contributed the mini review containing the biomaterials that have been reported with efficient promotion to gas hydrate formation, such as, lignosulfonates, amino acids, biosurfactants, and biological porous structures, among which, amino acids, with the high species diversity, attracted the most attention and exhibited the best prospects. Besides the studies with chemicals as kinetic promoters as mentioned above, Uchida et al. reported the propane hydrate formation with nanobubble-containing water and we can also view it as using nanobubbles as kinetic promoters, which was approved as a novel technology to kinetically enhance hydrate formation.

Besides kinetic promoters, thermodynamic accelerating agents are also focused in this research topic. Miyamoto et al., with the aim of using hydrates as phase change materials for thermal storage, adopted tetrabutylphosphonium oxalate (TBPOx) as the hydrate former for semiclathrate hydrate formation and determined the phase equilibrium temperature and the dissociation heat, via which, TBPOx were confirmed as an excellent hydrate former for hydrate-based thermal storage. Seol et al. introduced three large guest molecules, oxabicyclic compounds, as the thermodynamic promoters for methane hydrate formation. Via spectroscopic investigation and equilibrium measurements, the authors declared the great potential of the three large guest molecules as alternates to conventional promoters. Nguyen et al. reported the hydrogen hydrate formation supported by both tetrahydrofuran (THF) and perchloric acid (HClO_4_), and found that the co-inclusion of HClO_4_ into the THF hydrates efficiently enhanced the insertion of hydrogen molecules into the hydrate cages, producing great application potential in hydrate-based hydrogen storage.

